# Electric vehicle charging dataset with 35,000 charging sessions from 12 residential locations in Norway

**DOI:** 10.1016/j.dib.2024.110883

**Published:** 2024-08-30

**Authors:** Åse Lekang Sørensen, Igor Sartori, Karen Byskov Lindberg, Inger Andresen

**Affiliations:** a SINTEF, Department of Architectural Engineering, P.O. Box 124 Blindern, 0314 Oslo, Norway; b Norwegian University of Science and Technology (NTNU), Department of Architecture and Technology, 7491 Trondheim, Norway; c Norwegian University of Science and Technology (NTNU), Department of Electric Energy, 7491 Trondheim, Norway

**Keywords:** Electric vehicle (EV) charging data, Residential case study, EV charging schedules, Energy use, EV charging power, EV battery capacity, Hourly EV battery state of charge (SoC), Energy flexibility

## Abstract

This data article refers to the paper “A method for generating complete EV charging datasets and analysis of residential charging behaviour in a large Norwegian case study” [1]. The Electric Vehicle (EV) charging dataset includes detailed information on plug-in times, plug-out times, and energy charged for over 35,000 residential charging sessions, covering 267 user IDs across 12 locations within a mature EV market in Norway. Utilising methodologies outlined in [1], realistic predictions have been integrated into the datasets, encompassing EV battery capacities, charging power, and plug-in State-of-Charge (SoC) for each EV-user and charging session. In addition, hourly data is provided, such as energy charged and connected energy capacity for each charging session. The comprehensive dataset provides the basis for assessing current and future EV charging behaviour, analysing and modelling EV charging loads and energy flexibility, and studying the integration of EVs into power grids.

Specifications Table

**Table:** 

Subject	Renewable Energy, Sustainability and the Environment.
Specific subject area	Electric Vehicle (EV) charging at residential locations, with related time schedules, energy loads, and shifting potential.
Type of data	CSV files, Tables
Data collection	Residential charging reports were collected from charge point operators and companies in Norway: Current Eco AS (for locations ASK, BAR, BER, BOD, OSL_1, OSL_2, OSL_S, TRO), Zaptec Charger AS (for location BAR_2), Kople AS (for location KRO), Mer Norway AS (for location OSL_T), and NTE Marked AS (location TRO_R). The charging reports are available for monitoring and billing purposes.
Data source location	Data from 12 residential locations in Norway: Asker (59.83 N, 10.43E), Bærum (2) (59.56 N, 10.30E), Bergen (60.23 N, 5.19E), Bodø (67.16 N, 14.24E), Krokkleiva (59.86 N, 10.78E), Oslo (4) (59.54 N,10.45E), and Trondheim (2) (63.44 N, 10.42E).
Data accessibility	Repository name: zenodo.org Data identification number: 10.5281/zenodo.12730566 [2] Direct URL to data: https://doi.org/10.5281/zenodo.12730566
Related research article	Å.L. Sørensen, I. Sartori, K.B. Lindberg, I. Andresen, A method for generating complete EV charging datasets and analysis of residential charging behaviour in a large Norwegian case study, Sustain. Energy, Grids Networks. 36 (2023). https://doi.org/10.1016/j.segan.2023.101195.

## 1 Value of the Data

• The global rise in Electric Vehicle (EV) adoption, anticipated to reach a projected 35 % sales share by 2030 [3], underscores the critical need for comprehensive data and research on EV charging.
• A significant gap exists in the availability of EV charging data necessary for detailed analyses and modelling of EV charging patterns and energy flexibility [4].
• Our dataset addresses this gap by providing detailed information on plug-in/plug-out times and energy charged from over 35,000 charging sessions across 12 residential locations in Norway, representing 267 diverse users within a mature EV market.
• By employing methodologies outlined in [1,5], we have integrated realistic predictions into the dataset, resulting in user-friendly EV charging datasets essential for a wide range of EV research.
• The predictions encompass battery capacities and charging power for each EV user, charging time, idle time, and plug-in State of Charge (SoC) for each charging session, as well as hourly data such as energy charged and connected energy capacity for each charging session.
• The comprehensive dataset provides the basis for a variety of energy studies, including analyses of EV charging behaviour, load forecasting, energy flexibility, and the integration of EV charging into power grids.

## 2 Background

The use of EVs is central in meeting emissions reduction goals outlined in the Paris Climate Change Agreement [6]. Globally, EVs held a 14 % market share in 2022, with Norway standing at 88 % [3]. The predominant use of home and workplace charging influences the energy and power loads of buildings [7]. However, EV charging can often be shifted in time without affecting user habits, making it a promising solution for utilizing energy flexibility. Consequently, smart charging is becoming an increasingly important topic [8], particularly in buildings and locations with limited grid capacity or excess solar photovoltaic (PV) electricity.

This data article refers to the paper “A method for generating complete EV charging datasets and analysis of residential charging behaviour in a large Norwegian case study” [1]. The study revealed a general lack of EV charging data essential for data-driven analyses and modelling of EV charging and flexibility. The case study in [1] included a dataset with >35,000 residential charging sessions, which is made openly accessible in this data article.

## 3 Data Description

This article describe the dataset of the case study in [1], available in the linked repository. It consists of four datasets (csv-files) as described in this section. In the data provided with this article, Central European Time (CET) zone is used, which is GMT +1. Daylight saving time (DST) applies.

**Dataset 1: Residential charging reports**

Dataset 1 consists of original charging report, as outlined in Table 1. The dataset describes plug-in/plug-out times and energy charged from 35.377 charging sessions across 12 residential locations. The data for location TRO_R is further described in [5,9].

**Table 1:** Dataset 1: Residential charging reports.

Data	Description
location	Location of the CP. Data is available from 12 residential locations: ASK: Asker (sub-urban), data from 2018 to 11–15 to 2020–02–03, 6372 sessions BAR: Bærum (sub-urban), data from 2018 to 09–07 to 2020–02–03, 1969 sessions BAR_2: Bærum (sub-urban), data from 2020 to 02–04 to 2021–08–06, 1028 sessions BER: Bergen sør-vest (urban), data from 2019 to 10–31 to 2020–02–03, 308 sessions BOD: Bodø (urban), data from 2018 to 10–24 to 2020–02–02, 508 sessions KRO: Krokkleiva (rural), data from 2021 to 01–15 to 2021–05–06, 492 sessions OSL_1: Oslo (urban), data from 2019 to 10–08 to 2020–02–02, 464 sessions OSL_2: Oslo (urban), data from 2019 to 11–25 to 2020–02–02, 127 sessions OSL_S: Oslo sør-øst (urban), data from 2018 to 02–06 to 2020–02–03, 9757 sessions OSL_T: Oslo Tveita (urban), data from 2019 to 11–15 to 2021–03–29, 5478 sessions TRO: Trondheim (urban), data from 2019 to 03–08 to 2020–02–03, 2168 sessions TRO_R: Trondheim Risvollan (urban), data from 2018 to 12–21 to 2020–01–31, 6706 sessions
session_id	Charging session ID (*n* = 35.377)
user_id	User ID (*n* = 267)
plugin_time	Plug-in date and time (format 2019–11–28 19:08:00)
plugout_time	Plug-out time (format 2019–11–28 19:08:00)
connection_time	Duration of the EV connection time [h], per charging session (decimal hours)
energy_session	Energy charged [kWh], per charging session

**Dataset 2: Predictions per user**

Dataset 2 consists of predictions per user, as outlined in Table 2.

**Table 2:** Dataset 2: Predictions per user.

Data	Description
user_id	User ID (*n* = 267)
charging_power	Predicted charging power [kW] per user ID (for 261 user_id)
battery_capacity	Predicted net battery capacity [kW] per user ID (for 261 user_id)

**Dataset 3: Predictions per charging session**

Dataset 3 consists of predictions per charging session, as outlined in Table 3, for all User IDs with predictions in Dataset 2. Dataset 3 can be merged with Dataset 1 (by session_id), to get the complete charging session data.

**Table 3:** Dataset 3: Predictions per charging session.

Data	Description
user_id	User ID (*n* = 261)
session_id	Charging session ID (*n* = 34.537)
charging_time	Duration of the EV charging time [h], per charging session (decimal hours)
SoC_diff	Difference in battery SoC per charging session [percent]
SoC_start	Battery start SoC per charging session [percent], with assumed end SoC of 95 %
idle_time	Duration of the non-charging idle time [h], per charging session (decimal hours)
idle_session	Idle energy capacity [kWh], per charging session
non_flex_session	Energy charged (energy_session) [kWh] for charging sessions with idle times less than 1 h

**Dataset 4: Hourly predictions per charging session**

Dataset 4 consists of hourly predictions per charging session, as outlined in Table 4.

**Table 4:** Dataset 4: Hourly predictions per charging session.

Data	Description
user_id	User ID (*n* = 261)
session_id	Charging session ID (*n* = 34.537)
date_from	Date and time (format 2019–11–28 19:00:00), where 19:00 represents 19:00 to 20:00
energy_charged_i	Energy charged [kWh] per hour, per charging session
energy_idle_i	Idle energy capacity [kWh] per hour, per charging session
energy_connected_i	Connected energy capacity [kWh] per hour, per charging session
SoC_diff_i	Difference in battery SoC per hour, per charging session [percent]
SoC_from_i	Battery start SoC per hour, with assumed end SoC of 95 % [percent]
SoC_to_i	Battery end SoC per hour, with assumed end SoC of 95 % [percent]

## 4 Experimental Design, Materials and Methods

This section offers a detailed account of the methods used for data acquisition, including data collection and cleaning for Dataset 1, user predictions for Dataset 2, and EV charging predictions for Dataset 3 and Dataset 4.

**Data collection and cleaning for Dataset 1**

The residential charging reports were collected from charge point operators (CPOs) and apartment buildings in Norway:

For locations ASK, BAR, BER, BOD, OSL_1, OSL_2, OSL_S, TRO:

• Data was provided by Current Eco AS [10], which develops Charge Point Management Systems.

For location BAR_2:

• Data was downloaded by a housing cooperation, using the EV charging portal from EV charging manufacturer Zaptec Charger AS [11].

For location KRO:

• Data was provided by the CPO Kople AS [12].

For location OSL_T:

• Data was provided by the CPO Mer Norway AS [13].

For location TRO_R:

• Data was provided by the CPO NTE Marked AS [14].

Data cleaning was performed, resulting in the 35.377 charging session IDs and 267 user IDs as described in Dataset 1 (Table 1). The data cleaning procedure is described in Table 5. Finally, time zone and daylight-saving time (DST) corrections were made before adding calendar data, such as weekdays.

**Table 5:** Data cleaning procedure for original charging reports.

Data cleaning criteria	Reason	Removed
No energy charged (≤0.5 kWh)	Assumed faulty sessions	2289 sessions
Too high energy charged (>150 kWh)	Assumed faulty sessions (maximum battery capacity for EVs is 100 kWh)	2 sessions
Connection time < 2 min	Assumed faulty sessions	131 sessions
Connection time > 5 days	Affects average connection and idle time	155 sessions
Average power > available charging power (≥11.5 kW). 1. Sessions with average power ≥11.5 kW	Incorrect values. Average power calculated as energy charged divided by connection time	40 sessions (plug-out times set to NA)
2. OSL_T sessions with average power ≥11.5 kW, though 22 kW available at shared CPs	Few sessions had an average power ≥11.5 kW, which falls outside the scope of the database	22 sessions (plug-out times set to NA)
3. Two user IDs were removed (all 338 sessions) due to multiple sessions with average power > 11.5 kW (*n* = 12 + 6)	Assumed that they normally used shared CPs with higher charging power; removing plug-out times for some sessions could mislead results	2 user IDs 338 sessions
User IDs with less than 10 charging sessions	Few sessions per User ID	72 user IDs 268 sessions

**User predictions for Dataset 2**

For predicting charging power and net battery capacity per user in Dataset 2 (Table 2), the following steps were taken [1].

***EV charging power (charging_power)***

1. *Assumption*: Each user ID has at least one session where the charger is unplugged while still charging.
2. *Calculation*: If unplugged during charging, the connection time equals the charging time. The average charging power (*P_charging_*) is calculated using Eqs. (1) to (3), with data from Dataset 1: plugout_time (*t_plug-out_*), plugin_time (*t_plug-in_*), and energy_session (*E_charged_*).
  CP connection time for an EV session:
  (1) $$t_{connection}=t_{plug-out}-t_{plug-in}$$
  When plug-out during charging:
  (2) $$t_{charging}=t_{connection}$$
  Average EV charging power:
  (3) $$P_{charging}=E_{charged}/t_{charging}$$
3. *Identifying key sessions:* The highest *P_charging_* value for each user ID is selected as the preliminary prediction (*P_preliminary_*).
4. *Filtering Errors and Outliers:* To improve accuracy, *P_preliminary_* is compared to typical EV charger capacities, categorized into three levels:
  ○ Level 1: <4 kW (PHEVs and earlier BEVs)
  ○ Level 2: 4–8 kW (Standard BEVs)
  ○ Level 3: 8–11.5 kW (Newer/larger BEVs)
5. *Validation:* If a user ID has at least two sessions within the same category, *P_preliminary_* is accepted as the final charging_power prediction (*P_user_*). Otherwise, the session is considered an outlier, and a new *P_preliminary_* is calculated. This process repeats until all user IDs have a final *P_user_*.
6. *Results*: For 267 user IDs:
  ○ 93 % (249 IDs) required no filtering.
  ○ 6 % (17 IDs) required one outlier removal.
  ○ 1 ID required two outlier removals.
  ○ 6 IDs with *P_user_* < 2 kW were removed due to market inconsistency (too low *P_user_*).

***Net battery capacity (battery_capacity)***

1. *Assumption*: Each user ID has at least one session where the EV battery is charged from a defined minimum to a defined maximum state of charge (SoC) level.
2. *Calculation*: The session with the highest energy_session (*E_charged_*) for each user is selected. This maximum energy value is multiplied by an efficiency factor (ɳ = 88 %) to calculate the approximate energy stored in the battery (*E_battery_*), as shown in Eq. (4).Maximum energy stored in battery:
  (4) $$\begin{array}{c} \;\text{max}\left(E_{\mathrm{batt}\mathrm{ery}}\right)=\text{max}\left(E_{\mathrm{char}\mathrm{ged}}\right)\times \eta \end{array}$$
  Battery capacity prediction:
  (5) $$\begin{array}{c} E_{\mathrm{batt}\mathrm{ery}-\mathrm{size}}=\text{max}\left(E_{\mathrm{char}\mathrm{ged}}\right)/\mathrm{So}C_{\mathrm{range}} \end{array}$$
  The calculated maximum energy values (Eq. (4)) are divided by an assumed SoC range for the charging session (Eq. (5)) to predict battery capacities. Two SoC ranges are used, depending on battery size:
  ○ Small/Medium (EV-SM): 10 % minimum SoC, 90 % SoC range.
  ○ Large (EV-L): 20 % minimum SoC, 80 % SoC range.

**EV charging predictions for Dataset 3 and Dataset 4**

For charging predictions in Dataset 3 (Table 3) and Dataset 4 (Table 4), the following steps were taken [1,5].

***Energy charged per hour (energy_charged_i)***

Energy charged during sessions is distributed hourly using the methodology from [5].

*Calculation*: The hourly charging loads (*E_load (i)_*) are calculated by multiplying the EV charging power prediction per user ID (charging_power, *P_user_*) with the hourly charging time, as shown in Eq. (6). It is assumed that charging starts immediately after plug-in and the charging power remains constant throughout the charging time.

Charging load hour i: $E_{load(i)}=P_{user}\times t_{charging\;(i)}$ where

(6) $$\sum E_{load\left(i\right)}=\;E_{charged}$$

***Duration of the EV charging time (charging_time) and non-charging idle time (idle_time)***

The duration of EV charging time and non-charging idle time is calculated using Eqs. (7) and (8).

EV charging time:

(7) $$t_{charging}=E_{charged}/P_{charging}$$

Idle time per session:

(8) $$t_{idle}=t_{connection}-t_{charging}$$

If the initial assumption of EV charging power (charging_power) is filtered during the charging power assumption, the EV charging time may exceed the connection time for some EV sessions. In such cases, the charging_time is designated as NA.

***Idle energy capacity (idle_session, energy_idle_i)***

Hourly values for idle energy capacities are predicted by using the methodology presented in [5].

*Calculation*: Hourly idle energy capacities for each session are calculated by multiplying the idle times by the assumed charging power for the user ID, as shown in the following equation.

Idle energy capacity hour i:

(9) $$E_{idle\left(i\right)}=P_{user}\times t_{idle\;\left(i\right)}$$

idle_session represents the sum of the hourly idle energy capacities per session.

***Connected energy capacity (energy_connected_i)***

The sum of the charging loads (*E_load (i)_*) and idle energy capacities (*E_idle (i)_*) is referred to as the connected energy capacity for each hour, as shown in the following equation.

Connected energy capacity for hour *i*:

(10) $$E_{connected\left(i\right)}=E_{load\left(i\right)}+E_{idle\left(i\right)}$$

***Non-flexible energy charged (non_flex_session)***

Charging sessions with idle times less than 1 h are defined as non-flexible [1].

***SoC values***

SoC values are predicted by using the methodology presented in [1].

SoC_diff_i: During each hour of a charging session, the SoC difference for each EV is computed as the hourly energy stored in the battery (energy load adjusted for efficiency) divided by the predicted battery capacity for each user (battery_capacity*, E_user-battery_*), as shown in the following equation.

SoC difference for hour i:

(11) $$SoC_{diff\left(i\right)}=E_{load\left(i\right)}\times ɳ/E_{user-battery}$$

SoC_diff: Represents the sum of the hourly SoC difference per session.

SoC_from_i and SoC_to_i: Assuming a final SoC value of 95 %, the SoC for each hour is iteratively determined starting from the final hour of each session and moving backward in time, hour-by-hour, until the initial session hour. For charging sessions where the predicted non-charging idle time is less than one hour, no final SoC values are assumed due to potential user interruption.

SoC_start: Represents the battery start SoC for each charging session, assuming an end SoC of 95 %.

## Limitations

For most EV users in the case study, the maximum CP charging power available was 7.4 kW (32 A). It was possible for the users to manually activate up to 11 kW on their CP, but only for the EV models which support IT 3-phase charging.

## Ethics Statement

The authors have read and follow the ethical requirements for publication in Data in Brief. The current work does not involve human subjects, animal experiments, or any data collected from social media platforms.

## Declaration of generative AI and AI-assisted technologies in the writing process

During the preparation of this work the authors used ChatGPT (OpenAI) in order to improve language and readability. After using this tool/service, the authors reviewed and edited the content as needed and take full responsibility for the content of the publication.

## CRediT Author Statement

**Åse Lekang Sørensen**: Conceptualization, Methodology, Investigation, Data curation, Writing - original draft, Writing - review & editing. **Igor Sartori, Karen Byskov Lindberg, Inger Andresen**: Conceptualization, Methodology, Supervision.

## Data Availability

Data files: Electric vehicle charging dataset with 35,000 charging sessions from 12 residential locations in Norway (Original data) (zenodo).
